# Editorial: Homeostatic and retrograde signaling mechanisms modulating presynaptic function and plasticity

**DOI:** 10.3389/fncel.2015.00380

**Published:** 2015-09-30

**Authors:** Jaichandar Subramanian, Dion Dickman

**Affiliations:** ^1^Picower Institute for Learning and Memory, Massachusetts Institute of TechnologyCambridge, MA, USA; ^2^Department of Neurobiology, University of Southern CaliforniaLos Angeles, CA, USA

**Keywords:** homeostatic plasticity, presynaptic adaptation, retrograde signaling, neurotransmitter release, neurological disease

Dynamic reorganization of neural circuits can occur through the selective strengthening of synapses between neurons that are coactive in response to the encoded information (Shatz, 1990). However, the positive feedback resulting from synaptic strengthening and neuronal coactivity can lead to the destabilization of neuronal networks. Thus, synapses must maintain the capacity for change necessary for learning and memory, yet constrain this inherently destabilizing flexibility to enable stable neural function throughout life. Evidence has emerged in recent years that activity within neural circuits can shape the synaptic properties of component neurons in a manner that maintains stable excitatory drive, a process referred to as homeostatic synaptic plasticity (Turrigiano and Nelson, 2000; Pozo and Goda, 2010; Davis, 2013). Potent and adaptive homeostatic mechanisms have been demonstrated in a variety of systems to modulate activity at the level of an individual neuron, synapse, circuit, or entire network, and dysregulation at some or all of these levels may contribute to neuropsychiatric disorders, intellectual disability, and epilepsy (Wondolowski and Dickman, 2013). Greater mechanistic understanding of homeostatic plasticity will provide key insights into the etiology of these disorders, which may result from network instability and synaptic dysfunction. Over the past 15 years, the molecular mechanisms of this form of plasticity have been intensely studied in various model organisms, including invertebrates and vertebrates (Davis and Müller, 2015). Though, once thought to have a predominantly postsynaptic basis, emerging evidence suggests that homeostatic mechanisms act on both sides of the synapse, through mechanisms such as retrograde signaling, to orchestrate compensatory adaptations that maintain stable network function (Vitureira et al., 2012). These trans-synaptic signaling systems ultimately alter neurotransmitter release probability by a variety of mechanisms including changes in vesicle pool size and calcium influx (Davis and Müller, 2015). These adaptations are not expected to occur homogenously at all terminals of a pre-synaptic neuron, as they might synapse with neurons in non-overlapping circuits. However, the factors that govern the homeostatic control of synapse-specific plasticity are only beginning to be understood.

The homeostatic mechanisms that regulate post-synaptic strength has been extensively studied and reviewed. In contrast, relatively little attention has been paid to pre-synaptic homeostatic plasticity mechanisms and retrograde signaling. This research topic is intended to shine light on this very important topic and we start this with a comprehensive review of different homeostatic plasticity mechanisms that govern neurotransmitter release probability (Lazarevic et al., 2013). Homeostatic adaptations are not merely functional changes that affect synaptic strength but involve a whole array of structural modifications in both excitatory and inhibitory neurons. A broad review on this topic is presented by Yin and Yuan (2014). This is followed by an in depth review on axonal adaptations in inhibitory neurons by Frias and Wierenga (2013).

Many forms of homeostatic plasticity, such as synaptic scaling, occur over a period of several hours to days. In contrast, activity dependent strengthening of synapses (commonly referred to as Hebbian plasticity) is thought to happen on shorter time scales. Computational analyses reveal that slow forms of homeostasis are inadequate to counter the runaway excitability caused by hebbian plasticity. The next two chapters report novel means for achieving rapid homeostasis in response to network activity. The paper by Delattre V et al. describes how the timing of network activity in relation to that of spikes can determine the direction of plasticity (Delattre et al., 2015). The paper by Faghihi and Moustafa shows how Hebbian plasticity and fast retrograde signaling can interact to generate highest efficiency to encode differences in input stimuli (Faghihi and Moustafa, 2015).

In order to adapt to activity perturbations, neurons should have the machinery to sense and respond in adaptive ways that will lead to the modification of appropriate pre-synaptic function. These signals can be intrasynaptic or trans-synaptic depending on their locus of production and action. The next two chapters will provide extensive reviews on the roles of a retrograde signaling molecule Nitric Oxide (Hardingham et al., 2013) and the role of voltage gated calcium channels Frank, 2014b) in mediating (Frank, 2014b) homeostatic adaptations. In the following chapter, Qiu et al., provide evidence for endocannabinoid receptors in generating long-term depression in Molecular layer I-Purkinjee cell synapses in the cerebellum (Bing et al., 2015).

Much of the progress in our understanding of the interplay of pre- and post-synaptic cells to maintain synaptic strength has come from detailed studies of the larval neuromuscular junction of *Drosophila melanogaster* (Frank, 2014a). At this model glutamatergic synapse, genetic or pharmacological perturbations to postsynaptic receptors initiate a robust homeostatic signaling system in which a retrograde signal induces a precise increase in presynaptic release that compensates for this perturbation, restoring normal muscle excitability. The speed, accessibility, and genetic tractability of the fly NMJ make this a particularly attractive model to study the homeostatic control of presynaptic release. Using this system, Ueda and Wu report the mechanisms by which postsynaptic excitability is kept constant despite an increase in pre-synaptic growth when the larvae are reared at high temperatures (Ueda and Wu, 2015). In order to harness the full potential of this system, Brusich et al. has developed a new genetic tool that has enabled them to identify different molecules needed for the long-term maintenance of homeostatic synaptic plasticity (Brusich et al., 2015).

The potential importance of homeostatic mechanisms to circuit stability has long been recognized. Recently, many neurological and neuropsychiatric disease susceptibility genes have been found to be important for different aspects of homeostatic signaling. Interestingly, many of these diseases are comorbid with epilepsy, a condition predicted to be a consequence of runaway excitation resulting from impaired negative feedback plasticity mechanisms. The final two chapters review important links between presynaptic homeostatic mechanisms and neurological disease. The minireview by Meier et al. focuses on the presynaptic mechanisms that may contribute to the etiology of epilepsy (Meier et al., 2014). We conclude this topic with an extensive review on neurological disease susceptibility genes and their role in homeostatic synaptic plasticity (Wondolowski and Dickman, 2013).

## Conflict of interest statement

The authors declare that the research was conducted in the absence of any commercial or financial relationships that could be construed as a potential conflict of interest.

## References

[B1] Bing Y. H. Wu M. C. Chu C. P. Qiu D. L. (2015). Facial stimulation induces long-term depression at cerebellar molecular layer interneuron-Purkinje cell synapses *in vivo* in mice. Front. Cell. Neurosci. 9:214. 10.3389/fncel.2015.00214 26106296 PMC4460530

[B2] Brusich D. J. Spring A. M. Frank C. A. (2015). A single-cross, RNA interference-based genetic tool for examining the long-term maintenance of homeostatic plasticity. Front. Cell. Neurosci. 9:107. 10.3389/fncel.2015.00107 25859184 PMC4374470

[B3] Davis G. W. (2013). Homeostatic signaling and the stabilization of neural function. Neuron 80, 718–728. 10.1016/j.neuron.2013.09.044 24183022 PMC3856728

[B4] Davis G. W. Müller M. (2015). Homeostatic control of presynaptic neurotransmitter release. Annu. Rev. Physiol. 77, 251–270. 10.1146/annurev-physiol-021014-071740 25386989

[B5] Delattre V. Keller D. Perich M. Markram H. Muller E. B. (2015). Network-timing-dependent plasticity. Front. Cell. Neurosci. 9:220. 10.3389/fncel.2015.00220 26106298 PMC4460533

[B6] Faghihi F. Moustafa A. A. (2015). The dependence of neuronal encoding efficiency on Hebbian plasticity and homeostatic regulation of neurotransmitter release. Front. Cell. Neurosci. 9:164. 10.3389/fncel.2015.00164 25972786 PMC4412074

[B7] Frank C. A. (2014a). Homeostatic plasticity at the Drosophila neuromuscular junction. Neuropharmacology 78, 63–74. 10.1016/j.neuropharm.2013.06.015 23806804 PMC3830618

[B8] Frank C. A. (2014b). How voltage-gated calcium channels gate forms of homeostatic synaptic plasticity. Front. Cell. Neurosci. 8:40. 10.3389/fncel.2014.00040 24592212 PMC3924756

[B9] Frias C. P. Wierenga C. J. (2013). Activity-dependent adaptations in inhibitory axons. Front. Cell. Neurosci. 7:219. 10.3389/fncel.2013.00219 24312009 PMC3836028

[B10] Hardingham N. Dachtler J. Fox K. (2013). The role of nitric oxide in pre-synaptic plasticity and homeostasis. Front. Cell. Neurosci. 7:190. 10.3389/fncel.2013.00190 24198758 PMC3813972

[B11] Lazarevic V. Pothula S. Andres-Alonso M. Fejtova A. (2013). Molecular mechanisms driving homeostatic plasticity of neurotransmitter release. Front. Cell. Neurosci. 7:244. 10.3389/fncel.2013.00244 24348337 PMC3847662

[B12] Meier J. C. Semtner M. Winkelmann A. Wolfart J. (2014). Presynaptic mechanisms of neuronal plasticity and their role in epilepsy. Front. Cell. Neurosci. 8:164. 10.3389/fncel.2014.00164 24987332 PMC4060558

[B13] Pozo K. Goda Y. (2010). Unraveling mechanisms of homeostatic synaptic plasticity. Neuron 66, 337–351. 10.1016/j.neuron.2010.04.028 20471348 PMC3021747

[B14] Shatz C. J. (1990). Impulse activity and the patterning of connections during CNS development. Neuron 5, 745–756. 2148486 10.1016/0896-6273(90)90333-b

[B15] Turrigiano G. G. Nelson S. B. (2000). Hebb and homeostasis in neuronal plasticity. Curr. Opin. Neurobiol. 10, 358–364. 10.1016/S0959-4388(00)00091-X 10851171

[B16] Ueda A. Wu C. F. (2015). The role of cAMP in synaptic homeostasis in response to environmental temperature challenges and hyperexcitability mutations. Front. Cell. Neurosci. 9:10. 10.3389/fncel.2015.00010 25698925 PMC4313691

[B17] Vitureira N. Letellier M. Goda Y. (2012). Homeostatic synaptic plasticity: from single synapses to neural circuits. Curr. Opin. Neurobiol. 22, 516–521. 10.1016/j.conb.2011.09.006 21983330 PMC3378479

[B18] Wondolowski J. Dickman D. (2013). Emerging links between homeostatic synaptic plasticity and neurological disease. Front. Cell. Neurosci. 7:223. 10.3389/fncel.2013.00223 24312013 PMC3836049

[B19] Yin J. Yuan Q. (2014). Structural homeostasis in the nervous system: a balancing act for wiring plasticity and stability. Front. Cell. Neurosci. 8:439. 10.3389/fncel.2014.00439 25653587 PMC4299450

